# Combination of Global Features for the Automatic Quality Assessment of Retinal Images

**DOI:** 10.3390/e21030311

**Published:** 2019-03-21

**Authors:** Jorge Jiménez-García, Roberto Romero-Oraá, María García, María I. López-Gálvez, Roberto Hornero

**Affiliations:** 1 Biomedical Engineering Group, University of Valladolid, Paseo de Belén 15, 47011 Valladolid, Spain; 2 Department of Ophthalmology, Hospital Clínico Universitario de Valladolid, Avenida Ramón y Cajal 3, 47003 Valladolid, Spain; 3 Instituto de Oftalmobiología Aplicada, University of Valladolid, Paseo de Belén 17, 47011 Valladolid, Spain; 4 Instituto de Investigación en Matemáticas (IMUVA), University of Valladolid, 47011 Valladolid, Spain; 5 Instituto de Neurociencias de Castilla y León (INCYL), University of Salamanca, 37007 Salamanca, Spain

**Keywords:** diabetic retinopathy, fundus images, retinal image quality assessment, Shannon entropy, spectral entropy, continuous wavelet transform, multilayer perceptron

## Abstract

Diabetic retinopathy (DR) is one of the most common causes of visual loss in developed countries. Computer-aided diagnosis systems aimed at detecting DR can reduce the workload of ophthalmologists in screening programs. Nevertheless, a large number of retinal images cannot be analyzed by physicians and automatic methods due to poor quality. Automatic retinal image quality assessment (RIQA) is needed before image analysis. The purpose of this study was to combine novel generic quality features to develop a RIQA method. Several features were calculated from retinal images to achieve this goal. Features derived from the spatial and spectral entropy-based quality (SSEQ) and the natural images quality evaluator (NIQE) methods were extracted. They were combined with novel sharpness and luminosity measures based on the continuous wavelet transform (CWT) and the hue saturation value (HSV) color model, respectively. A subset of non-redundant features was selected using the fast correlation-based filter (FCBF) method. Subsequently, a multilayer perceptron (MLP) neural network was used to obtain the quality of images from the selected features. Classification results achieved 91.46% accuracy, 92.04% sensitivity, and 87.92% specificity. Results suggest that the proposed RIQA method could be applied in a more general computer-aided diagnosis system aimed at detecting a variety of retinal pathologies such as DR and age-related macular degeneration.

## 1. Introduction

Diabetic Retinopathy (DR) is a visual complication of diabetes. Due to the high prevalence of this disease, DR has become one of the most common causes of blindness in developed countries [1]. The symptoms related to DR are not perceived by patients until the advanced stages of the disease, when the treatment is less effective and the risk of visual loss is high. Therefore, screening programs are essential to diagnose DR in its early stages and prevent blindness [2]. Retinal imaging is very useful in clinical applications aimed at detecting several diseases that affect the retina, such as DR, age-related macular degeneration (AMD), glaucoma, etc. [1,3]. Fundus images are color photographs of the retina acquired with a fundus camera. In these photographs, retinal structures like the optic disk (OD), the macula, and the blood vessels can be imaged with high detail [4]. Periodic exams of the retina using fundus images are one of the most accepted techniques to assess the presence and severity of DR [1,2]. However, due to the high prevalence of diabetes, a large number of fundus images needs to be analyzed by ophthalmologists [1,4]. Automated analysis of retinal images can help ophthalmologists to develop screening programs aimed at detecting DR by reducing the workload of specialists and increasing the cost-effectiveness [1]. Technical improvements have allowed an easier operation of fundus cameras, but the technical skills and the experience of the operator are directly related to the quality of the acquired images [1,3,4,5]. When operators obtain images of poor quality, retinal structures and possible lesions related to DR are not clearly visible. Poor quality images cannot be graded unless they are acquired again [5]. The most frequent causes of ungradable fundus images are inadequate focus, blurring, or insufficient illumination [3,6,7,8]. Some large-scale studies reported an ungradable image rate of 10–20% due to insufficient quality [3,9,10]. Objective and automatic quality assessment of fundus images should be performed before they are analyzed by automatic systems or human graders. In the case of automatic systems aimed at detecting retinal pathologies, an image quality assessment method is needed to prevent inaccurate diagnosis [5]. Thus, it is important to include an image quality assessment method as the first step in automatic retinal image analysis algorithms [1].

Retinal image quality assessment (RIQA) has received substantial attention during the last years. RIQA methods can be categorized as structural methods and generic methods [5,8,11,12]. Structural methods include segmentation-based methods to assess image quality. Many of them rely on the detection of blood vessels for quality assessment. Some approaches were based on the area of segmented vessels [13], or on the presence of small vessels around the macula [14]. Other authors divided the image into clusters that represented the background and image structures (including vessels and OD) [15]. Quality assessment was then performed using a support vector machine (SVM) classifier [15]. Fleming et al. used a vessel enhancement operator to analyze areas of clearly visible vessels [8]. Other studies employed local vessel density to assess the presence of segmented vessels in different regions of the image [16]. A more recent approach analyzed the area, fragmentation, and complexity of the vascular network in combination with SVM for image quality assessment [11]. Contrarily to structural approaches, generic methods avoid the segmentation stage [12]. Some previous generic approaches were based on histogram analysis and edge detection [17,18]. Lin et al. focused on the calculation of edge widths to assess the quality of fundus images [19]. Other studies employed local sharpness and illumination measures [6]. Entropy, texture, and the analysis of blurred areas have been also investigated [20,21,22,23]. Anisotropy-related focus measures based on the Rényi entropy and the Discrete Cosine Transform (DCT) have been also studied [24,25]. The work by Pires Dias et al. combined color, focus, contrast, and illumination measures to train a multilayer perceptron (MLP) neural network [12]. More recent studies focused on the analysis of illumination, homogeneity, saturation, and sharpness using the wavelet transform [26,27]. Other authors combined features related to image focus using the wavelet transform, Chevyshev moments, and a median filter-based statistical measure [28]. It should be noted that some approaches combined generic and structural methods, resulting in hybrid methods. Some authors complemented a previous clustering approach [15] using texture features to improve the results [5]. In other approaches, retinal blood vessels, OD, and macula were segmented, and features related to form, texture, and intensity were extracted to assess image quality [29]. Shao et al. combined OD localization with the illumination level and a general-purpose quality evaluator to perform RIQA [7]. Recent studies have focused on deep learning approaches, using the pretrained “*AlexNet*” and “*Inception v3*” convolutional neural networks for image quality assessment [30,31].

Structural and hybrid methods are limited by segmentation algorithms, that are usually inaccurate and error prone [5,12]. Generic methods achieved good performance while they are computationally simpler, as they are generally based on illumination or sharpness metrics [6,12]. Regarding to these metrics, recent RIQA methods combined wavelets and alternative color models [26,27]. Nevertheless, a common problem of generic methods is that they are based on simple quality features and their results do not usually agree with human graders [22]. Therefore, more accurate image quality measurements are needed. Previous research applied general-purpose quality metrics to develop generic RIQA methods [21,22]. These approaches considered the retinal images of inadequate quality as distorted images [22]. Recently, general-purpose no-reference image quality assessment (NR-IQA) methods inspired by the Natural Scenes Statistics (NSS) approach have shown promising results for image quality assessment [32,33,34,35]. Among the most popular NSS-based NR-IQA methods, natural images quality evaluator (NIQE) [34] and spatial and spectral entropy-based quality (SSEQ) [35] have gained relevance in the last years. NIQE and SSEQ quality-aware features are being used to build robust NR-IQA methods in a variety of applications [36,37]. However, to the best of our knowledge, only one NSS-based NR-IQA method has been successfully applied in the context of retinal image analysis [7].

Based on the aforementioned considerations, we hypothesized that combining NSS-based NR-IQA methods with generic features based on sharpness and luminosity can be useful to assess the quality of fundus images. Novel quality features related to NR-IQA methods, sharpness and luminosity could enhance the performance of generic RIQA methods. NIQE and SSEQ features can provide information about perceived image quality. Moreover, sharpness and luminosity features can be specifically designed for the characteristics of retinal images. Based on our preliminary work [38], the objective of this study was to combine novel generic methods to develop a RIQA method that could be useful in a more general retinal image analysis system. Features were extracted using NIQE and SSEQ methods. Additionally, sharpness and luminosity of retinal images were assessed using the continuous wavelet transform (CWT) and the hue saturation value (HSV) color model. These features were subsequently classified using a MLP neural network.

## 2. Retinal Image Database

**Figure 1:**
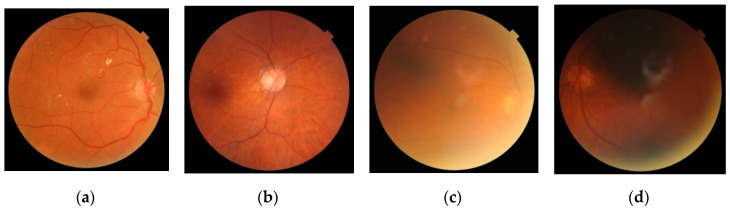
Examples of retinal images from the database. (**a**,**b**) Were labeled as adequate quality images, while (**c**,**d**) were considered of inadequate quality.

Our database was composed of 2107 fundus images from 688 patients. Images were provided by the “Instituto de Oftalmobiología Aplicada” of the University of Valladolid (Valladolid, Spain) and the “Hospital Clínico Universitario de Valladolid” (Valladolid, Spain). All subjects gave their informed consent to participate in the study. The study was conducted in accordance with the Declaration of Helsinki, and the protocol was approved by the Ethics Committee of the “Hospital Clínico Universitario de Valladolid” (PI 16-459). All images were captured using the Topcon TRC-NW400 (Topcon Medical Systems, Inc., Oakland, NJ, USA) fundus camera with 45° circular field of view (FOV) and were stored using the 24-bits color JPEG file format. Image size was 1956 × 1934 pixels, and the diameter of FOV was around 1850 pixels. Two images were captured per eye—A macula-centered image and an OD-centered image. Two experienced ophthalmologists decided whether each image had enough quality to be analyzed or not. Four criteria were considered by experts to assess image quality: (i) The OD borders were well defined, (ii) the blood vessels (especially the main arcades) were well defined, (iii) the retinal parenchyma (i.e., retinal nerve fiber layer) was visible, and (iv) the macula was distinguishable. In general, an image was considered to have an adequate quality when all requirements were met. Based on these criteria, 1810 out of the 2107 images were considered as adequate quality images, while the remaining 297 images had inadequate quality. Examples of images with different quality levels in our database are shown in Figure 1.

All the images in the database were randomly divided in two subsets—training and test sets. 50% of the images (1053 images, 905 of adequate quality and 148 of inadequate quality) were assigned to the training set. The remaining images (1054 images, 905 of adequate quality and 149 of inadequate quality) composed the test set.

## 3. Methods

Our methodology was composed of four stages. First, a preprocessing stage was implemented to adapt the images for subsequent processing. After preprocessing, several generic features were extracted for each image. Features were obtained by applying quality measurements based on image processing methods. Then, a feature selection algorithm was applied to find a subset of relevant and non-redundant features. Finally, a MLP neural network was trained using this reduced subset of features to assess the quality of images.

### 3.1. Preprocessing

A preprocessing stage was implemented to improve the performance of the algorithms that were developed in the subsequent stages of the proposed method. It has been observed that these algorithms produce border effects around the FOV that reduce the performance of quality assessment methods. Besides, pixels outside the FOV should not be considered for quality assessment because they do not belong to the retinal area. The preprocessing algorithm proposed in this study first locates the circular region that represents the FOV by estimating its diameter and center [39]. The FOV diameter was estimated using the intensity profile along one diagonal of the image. Then, the circular Hough transform was used to find the FOV center [39]. With these two parameters, a circular mask (*M_FOV_*) of the FOV was generated.

**Figure 2:**
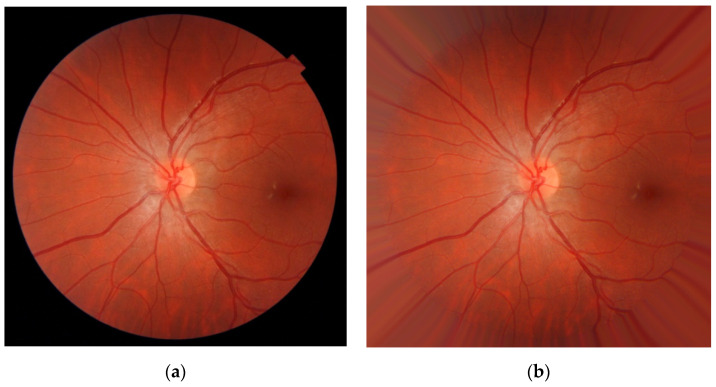
Result of the preprocessing stage: (**a**) Input image (*I_ORIG_*), (**b**) preprocessed image (*I_PREP_*).

An iterative FOV extension algorithm was subsequently implemented to reduce the influence of border effects near the FOV boundaries [40]. It was based on iteratively creating a preprocessed image, *I_PREP_*, derived from the original image, *I_ORIG_*, and an extended FOV mask, *M_EXT_*. In the first step of the algorithm *I_PREP_* = *I_ORIG_* (Figure 2a) and *M_EXT_* = *M_FOV_*. Then, for each iteration of the algorithm (*i*), the following operations were performed [40]:

1. The FOV mask border was extended using a dilation operator over *M_EXT_* (a 4-neighborhood diamond-shaped structuring element was used). This way, the FOV was enlarged to include new pixels around its border.
2. The values of *I_PREP_* corresponding to the new border pixels of *M_EXT_*(*i*) were substituted with the average value of the neighbor pixels in *I_PREP_* inside the mask *M_EXT_*(*I* − 1).
3. The FOV mask *M_EXT_* was updated with *M_EXT_*(*i*).

This workflow was repeated iteratively until all the pixels of the image were included in *M_EXT_*. This algorithm was applied on each channel of the red-green-blue (RGB) color model for color processing. An example of the result of the preprocessing stage can be seen in Figure 2b. The preprocessed image *I_PREP_* has no border that delimits the FOV. It should be noted that the pixels inside the FOV remained unaltered, while contrast between the aperture and the surrounding area was reduced.

### 3.2. Feature Extraction

Quality features were extracted using different image processing techniques. Two of them were general-purpose NSS-based NR-IQA methods originally developed to assess image quality in natural and distorted images. Two additional techniques focused on sharpness and illumination metrics were specifically designed for fundus images.

#### 3.2.1. Features Based on Spatial and Spectral Entropies

Entropy-based evaluation is one of the most extended techniques to assess information content in a system. As a consequence, it has been explored in different contexts, including biomedical signal processing [41,42], strategic decision making [43], and image quality assessment [35,37,44]. SSEQ is a NR-IQA method based on features related to spatial and spectral entropies in small non-overlapped regions (blocks) of the image [35]. The SSEQ feature extraction approach consisted of three stages [35]. In the first stage, three input images were obtained—the green channel of *I_PREP_* and 2 rescaled versions of the same image obtained with a down-sampling method using bicubic interpolation [35]. This procedure enabled us to perform a multi-scale analysis [35]. In this study, scale factors 1, 1/2, and 1/3 were used to obtain representations of the image with different sizes (scale 1: 1956 × 1934 pixels; scale 2: 978 × 967 pixels; scale 3: 652 × 645 pixels) [35]. Subsequently, images were divided into blocks of size *M* × *M* pixels. Spatial entropy (*SpacEn*) was then calculated for each block as the Shannon’s entropy [35]:

(1) $$SpacEn=-\underset{i}{\sum }p\left(i\right)\cdot \log_{2}p\left(i\right),$$

where *p*(*i*) represents the relative frequency of the intensity level *i* in a single block.

Spectral entropy (*SpecEn*) was also computed for each block. To obtain a representation in the frequency domain for image blocks, the 2-D DCT was used. DC (zero-frequency) component was excluded, and a normalized power spectral density (PSD) was obtained as [35]:

(2) $$P\left(k,l\right)=\frac{{\left[C\left(k,l\right)\right]}^{2}}{\sum_{k}\sum_{l}{\left[C\left(k,l\right)\right]}^{2}},\;\left(k,l\right)\neq \left(0,0\right),$$

where (*k*, *l*) are the indices of the DCT coefficients, *C*(*k*,*l*) is the DCT, and *P*(*k*,*l*) is the normalized PSD. *SpecEn* was then calculated for each block as the Shannon’s entropy of the normalized PSD [35]:

(3) $$SpecEn=-\underset{k}{\sum }\underset{l}{\sum }P\left(k,l\right)\cdot \log_{2}P\left(k,l\right).$$

After calculating *SpacEn* and *SpecEn* of all blocks, values between percentiles 15% and 85% were selected for both entropies. This way, only the central part of each distribution was analyzed and *SpacEn* and *SpecEn* were less sensitive to outliers [35]. Finally, the mean and the skewness of both entropies through all the selected blocks were calculated obtaining the features *SpacEn_MEAN_*, *SpacEn_SKEW_*, *SpecEn_MEAN_*, and *SpecEn_SKEW_* [35]. This process was repeated for the rescaled images. Hence, 12 features were extracted using the SSEQ method (2 mean values and 2 skewness values for each of the 3 scales). It should be noted that the block size *M* must be fixed. Although *M* is not a critical parameter [35], it has been selected as 1/60 of the image size in previous studies [35]. Following this approach, we set *M* = 32 pixels according to the size of the images in the training set.

#### 3.2.2. Features Based on Naturalness

The NIQE is a NR-IQA method based on the comparison of an image with a reference model of adequate quality images [34]. This reference model describes the characteristics of images of adequate quality. Thus, prior knowledge about the distortions induced by the acquisition process is not required to build the reference model [34]. Adequate quality images are expected to have similar features to those in the reference model, while differences are greater in distorted images [34]. Image quality assessment using NIQE was performed by comparing two NSS models—one that represented the reference model and another that characterized the image to be assessed [34].

In this study, we built a reference NSS model using the adequate quality fundus images in the training set of our database. For this task, a set of parameters was extracted from the blocks in these images. The process to obtain the reference NSS model is as follows [34]:

1. The image *I_PREP_* was normalized. The local mean *μ*(*x*, *y*) was subtracted for each pixel (*x*, *y*) and the result was divided by the local standard deviation *σ*(*x*, *y*) [34]:
  (4) $$I_{NORM}\left(x,y\right)=\frac{I_{PREP}\left(x,y\right)-\mu \left(x,y\right)}{\sigma \left(x,y\right)+1}.$$
2. The image *I_NORM_* was divided into blocks of size *P* × *P* pixels. Then, a subset of all the blocks in the image was selected based on the amount of local sharpness, *δ*(*b*), in each block *b* [34]. Blocks that exceeded a minimum amount of sharpness, *δ_MIN_*, were retained [34]:
  (5) $$\delta_{MIN}=T\cdot \underset{b}{\max}\delta \left(b\right),$$
  where *T* is a threshold between 0 and 1.
3. Each of the selected blocks was subsequently characterized by a zero-mean generalized Gaussian distribution (GGD). The parameters of shape (*α*) and spread (*β*) from the GGD were estimated for each block. Additionally, in each of the selected blocks, the products between adjacent pixels along 4 directions were calculated and characterized by four asymmetric generalized Gaussian distributions (AGGD). In this case, the estimated parameters from each of the AGGDs were the shape (*γ*), the left and right spreads (*β_l_*, *β_r_*), and the mean of the distribution (*η*). The process was repeated with a rescaled version of the same image in order to perform multi-scale analysis (978 × 967 pixels). A total of 36 parameters characterize each block—2 from the GGD (*α*, *β*) and 16 from 4 AGGDs (*γ*, *β_l_*, *β_r_*, and *η* in the 4 directions) using 2 scales.
4. Steps 1–3 were repeated for each image used to build the reference model.
5. The parameters from selected blocks in all the images were fitted to a 36-D multivariate Gaussian (MVG) model. The MVG probability distribution is defined as [34]:
  (6) $$f_{X}\left(x\right)=\frac{1}{{\left(2\pi \right)}^{k/2}{\left|\Sigma \right|}^{1/2}}e^{-\frac{1}{2}{\left(x-\nu \right)}^{T}\Sigma^{-1}\left(x-\nu \right)},$$
  where the vector **ν** and the covariance matrix **Σ** define the MVG model [34]. In this work, the parameters of the reference NSS model were denoted by **ν_M_** and **Σ_M_**.

The process to obtain the NSS model that characterizes the image to be assessed was analogous to the process for building the reference model. The only difference is that only the image under study, rather than a set of images, was used in this case. The NSS model of the image to be assessed is another MVG model defined by the parameters **ν_I_** and **Σ_I_**.

The NIQE quality index (*Q_NIQE_*) of the considered image was obtained by calculating the distance [34]:

(7) $$Q_{NIQE}=\sqrt{{\left(\nu_{M}-\nu_{I}\right)}^{T}{\left(\frac{\Sigma_{M}+\Sigma_{I}}{2}\right)}^{-1}\left(\nu_{M}-\nu_{I}\right)}.$$

In Formula (7), it can be seen that a low value of *Q_NIQE_* is associated to an image that resembles the reference model [34]. Therefore, this quality index can be considered a measure of the naturalness of an image.

It should be noted that two parameters must be fixed to build the NSS models—the block size *P* and the threshold *T*. Previous studies proposed that values of *P* between 32–160, as well as values of *T* between 0–1, were adequate for quality assessment [34]. For our images, we empirically found that the greater differences between adequate quality and inadequate quality images in the training set were obtained with *P* = 64 pixels and *T* = 0.1, which is consistent with previous studies [35].

#### 3.2.3. Features Based on the Continuous Wavelet Transform

Another set of features in this study were derived from the CWT. This technique has been widely used in image analysis and segmentation tasks [40,45,46]. The 2D CWT decomposes the image *I_PREP_* into several representations related with a scale factor *s*. It is defined as [47]:

(8) $$T_{\Psi }\left(I_{PREP},b,\theta ,s\right)=C_{\Psi }^{-\frac{1}{2}}\cdot \frac{1}{s}\int \Psi^{*}\left(\frac{r_{-\theta }\left(x-b\right)}{s}\right)I_{PREP}\left(x\right)d^{2}x,$$

where Ψ represents the mother wavelet, *r_−θ_* is the rotation operator by the angle *θ*, **x** = (*x*, *y*) are the coordinates of *I_PREP_* being considered, **b** is the translation of Ψ, and * represents the complex conjugate. *C_Ψ_* is a normalization constant.

Many different waveforms could be used as mother wavelet. In this study, the Mexican hat mother wavelet was selected because it is suitable for the detection of sharp edges in medical images [45,48]. In fundus images, the sharpness of the vessels and the OD boundaries has been also assessed using this mother wavelet [40,45]. It can be defined as [48]:

(9) $$\Psi_{MH}\left(x,y,s\right)=\frac{1}{\pi \cdot s^{4}}\left(1-\frac{x^{2}+y^{2}}{2s^{2}}\right)e^{-\frac{x^{2}+y^{2}}{2s^{2}}}.$$

**Figure 3:**
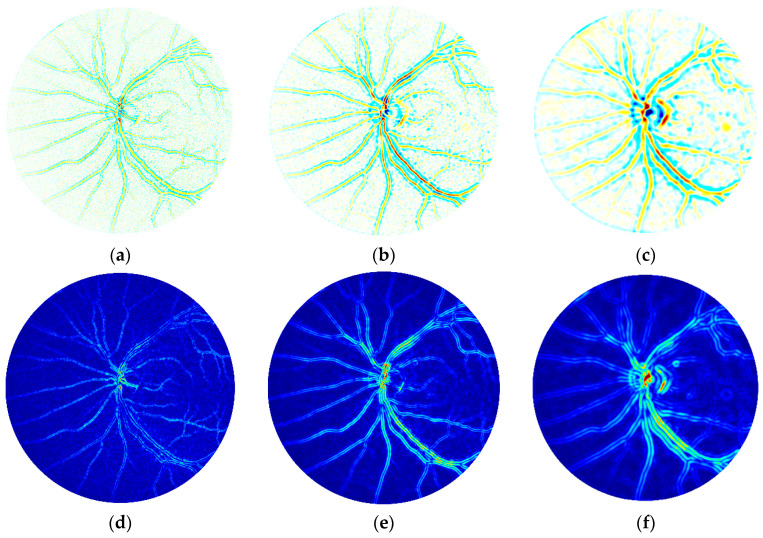
(**a**–**c**) Continuous wavelet transform *T*_Ψ_(*s*) representations at scales *s* = 4, 8, 16, respectively. (**d**–**f**) Local variance maps corresponding to the images in the first row.

The CWT was applied to the green channel of *I_PREP_* at scales *s* = 2, 4, 8, 16, 32 and 64. These scales were specifically selected to detect the borders of retinal structures (blood vessels, OD, and macula) [15,49]. Examples of the representations at *s* = 4, 8, and 16 are shown in Figure 3a–c. From the CWT representation obtained using *Ψ_MH_*, the variability of *T*_Ψ_(*s*) was calculated using the Shannon’s entropy (*ENT_CWT_*) as [26]:

(10) $$ENT_{CWT}\left(s\right)=-\underset{T_{\Psi }\left(s\right)}{\sum }p\left(T_{\Psi }\right)\cdot \log_{2}p\left(T_{\Psi }\right).$$

It has been observed that the amplitude of *T*_Ψ_(*s*) around the vessels and the OD was directly related to the sharpness of their edges (see Figure 3, top row). Thus, the CWT can be useful to identify if the retinal structures are clearly visible in the images. We assessed edge sharpness by calculating the local variance of *T*_Ψ_(*s*) [50]. For this task, standard deviation filters were applied to *T*_Ψ_(*s*) and the distributions of the obtained local variance maps, *σ*(*x*, *y*, *s*), were analyzed [50,51]. Since different scales of the CWT emphasize the borders of objects of different sizes, a circular standard deviation filter with radius *s* was selected for each scale [50]. Therefore, a circular filter with radius *r* = *s* was selected. Examples of local variance maps are shown in Figure 3d–f. For each scale, the mean (*MEAN_CWT_*) and the standard deviation (*SD_CWT_*) of *σ*(*x*, *y*, *s*) were computed:

(11) $$MEAN_{CWT}\left(s\right)=\frac{1}{N}\underset{\left(x,y\right)\in FOV}{\sum }\sigma \left(x,y,s\right),$$

(12) $$SD_{CWT}\left(s\right)=\sqrt{\frac{1}{N-1}\underset{\left(x,y\right)\in FOV}{\sum }{\left[\sigma \left(x,y,s\right)-CWT_{MEAN}\left(s\right)\right]}^{2}}.$$

In (11) and (12), *N* is the number of pixels inside the *FOV*.

#### 3.2.4. Luminosity Features

Sometimes, retinal images do not have enough quality because they were captured with insufficient or uneven illumination. In these cases, darkened areas in the image appear and they may hide retinal lesions [6,7,22]. Therefore, luminosity features can be useful to identify poorly illuminated images. Although the RGB color model has been used in the previous stages of the proposed method, it is more appropriate to use other color models when luminosity is to be assessed [7,20,22]. In this study, the HSV color model was used because differences between light and dark areas are more properly represented in the luminosity (*V*) channel [52]. Besides, color information can be separated from luminosity using this color model [52]. The conversion between RGB and HSV color models is simple and this color space is more similar to human perception of the color [51]. The illumination assessment method focused on the extraction of the background of the image using the *V* color channel. Noise was removed using a median filter with a square neighborhood *N*(*x*,*y*) of size 5 × 5 pixels in order to obtain a filtered image, *V_MED_*, as [51]:

(13) $$V_{MED}\left(x,y\right)=\underset{\left(i,j\right)\in N\left(x,y\right)}{\mathrm{median}}V\left(i,j\right).$$

**Figure 4:**
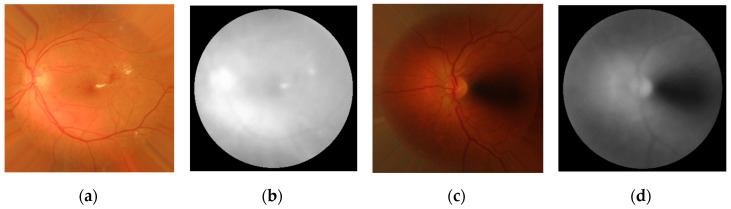
Examples of the extracted background using the *V* channel: (**a**) Adequate quality image and (**b**) its background; (**c**) inadequate quality image and (**d**) its background.

Then, the background of the images, *B*(*x*, *y*), was extracted using a large Gaussian filter, *g*(*x*, *y*), in order to attenuate vessels and subtle dark lesions [51]:

(14) $$B\left(x,y\right)=\overset{4\sigma }{\underset{i=-4\sigma }{\sum }}\overset{4\sigma }{\underset{j=-4\sigma }{\sum }}g\left(i,j\right)\cdot V_{MED}\left(x-i,y-j\right),$$

where *σ* is the standard deviation of *g*(*x*, *y*). The value of *σ* should be large enough to estimate the background and remove almost all vessels and dark lesions [53]. We fixed *σ* = 19 empirically according to the size of images in our training set. Examples of the extracted *B*(*x*, *y*) are shown in Figure 4.

Subsequently, *B*(*x*, *y*) was analyzed to obtain the luminosity level of its darkest zones. For this task, we calculated the luminosity values corresponding to the percentiles 1% (*Lum*_1_), 5% (*Lum*_5_), 10% (*Lum*_10_), 15% (*Lum*_15_), and 20% (*Lum*_20_) of the intensities in *B*(*x*, *y*). For images with a lighter and more uniform background (see Figure 4a,b) these percentiles would be higher than for images where darker background areas appear (Figure 4c,d). It should also be noted that *B*(*x*, *y*) may not be uniform in all images, especially when images were not captured with an adequate illumination. For this reason, the differences between consecutive luminosity percentiles were also calculated (*Lum*_5–1_, *Lum*_10–5_, *Lum*_15–10_, and *Lum*_20–15_). These differences can represent intensity variations along the background.

### 3.3. Feature Selection: Fast Correlation-Based Filter

A total of 40 features were finally extracted for each image. When a large set of features for a specific problem is considered, some of them may be redundant or irrelevant [54,55]. It is also important to note that a large number of features may lead to overfitting and reduce the ability of classifiers to make predictions on new data [55]. Feature selection algorithms try to overcome these difficulties by obtaining a reduced and optimum subset of features for a certain problem [55].

The fast correlation-based filter (FCBF) feature selection algorithm was used to identify relevant and non-redundant features [56]. This is a classifier-independent method with two stages. In the first stage, features are ordered according to their relevance. In the second stage, redundant features are removed. FCBF uses symmetrical uncertainty (*SU*) to assess both relevance and redundancy. It is defined as [56]:

(15) $$SU\left(X_{i}|X_{j}\right)=2\frac{H\left(X_{i}\right)-H\left(X_{i}|X_{j}\right)}{H\left(X_{i}\right)+H\left(X_{j}\right)},$$

where *H*(*X_i_*) is the Shannon entropy of the feature *X_i_*, and *H*(*X_i_*|*X_j_*) is the Shannon entropy of the feature *X_i_* after the observation of the feature *X_j_*.

The relevance of feature *X_m_* is defined as the *SU* between the class *C* (in this case, image quality) and *X_m_*. In the same way, redundancy is defined as the *SU* between pairs of features (*X_m_* and *X_n_*). Feature *X_m_* is considered redundant with *X_n_*, and thus removed, if [56]:

(16) $$SU\left(X_{n}|C\right)\geq SU\left(X_{m}|C\right),\;\mathrm{and}\;SU\left(X_{m}|X_{n}\right)\geq SU\left(X_{m}|C\right).$$

In order to improve the robustness of the feature selection process, a bootstrapping procedure was implemented [57]. Instances from the training set were randomly selected using the sampling with replacement technique to form bootstrap replicates [57]. For each replicate, instances from the training set were sampled with uniform probability until the original training set size was reached. This way, repeated instances were allowed [57]. We formed 1000 bootstrap replicates and applied the FCBF algorithm to each one. Features that were selected on at least half (500) of the runs formed the final optimum subset. Using bootstrapping, the feature selection stage was less dependent on the particularities of training set data [54].

### 3.4. Classification: Multilayer Perceptron Neural Network

Once a reduced subset of selected features was obtained, we employed a MLP neural network to classify the images into two categories—adequate quality and inadequate quality. MLPs are feed-forward networks in which the neurons are arranged in layers [58]. MLP neural networks have been widely used in classification tasks in the field of retinal image processing [12,53,59].

A three-layer MLP network (input, hidden, and output layers) was implemented in this study to solve the classification task [58]. The input layer had a number of neurons equal to the number of selected features. The output layer had only one neuron to perform binary classification [57,58]. The number of hidden neurons (*N_HIDDEN_*) was experimentally obtained to optimize the classification performance [57,60]. Hyperbolic tangent sigmoid activation function was used in the hidden layer, which accelerates the learning process of the network [58]. The logistic sigmoid activation function was selected in the output neuron since it is defined in the range (0–1) and, consequently, MLP outputs can be interpreted as posterior probabilities [58].

The training process of the MLP was aimed at minimizing an error function. We selected a cross-entropy error function for the minimization process [58]. Additionally, the scaled conjugate gradient was used for optimization in this study, since it generally converges faster than other techniques [58]. It is necessary to note that MLP training may result in overfitting, leading to errors when the network is tested on new data. To overcome this problem, weight decay regularization was implemented during training [60]. This technique redefines the error function by adding a penalty term that increases as the magnitude of weights increases [60]. The modified error function depends on a regularization parameter (*η*) that balances the contribution of the cross-entropy error function and the sum of the squares of the weights [60].

As explained in Section 2, the number of adequate quality images in our database was around 6 times greater than the number of inadequate quality images. In order to deal with class imbalance during training, we increased the number of instances corresponding to inadequate quality images using the synthetic minority oversampling technique (SMOTE) method [61]. This method creates synthetic instances of the data, **w**, by combining the *k* nearest neighbors of each sample in the minority class. Each synthetic sample is placed between the sample **u** and one of their nearest neighbors, **v**. Therefore, **w** is a linear combination of **u** and **v** [61]:

(17) w=u+c·(v−u)=c·v+(1−c)·u,

where *c* is a random number between 0 and 1. The number of synthetic samples depends on the number of neighbors (*k*) [61]. We set *k* = 5 in the training stage to obtain 740 synthetic minority class training samples. Thus, the training set was finally comprised of 905 adequate quality instances and 888 inadequate quality instances.

## 4. Results

### 4.1. Performance Evaluation

We used a MLP neural network to classify the features in two classes—adequate-quality and inadequate-quality images. The MLP configuration (*N_HIDDEN_* and *η*) had to be optimized in the training stage. We considered values of *N_HIDDEN_* ranging from 1 to 100 and values of *η* ranging from 0 (no regularization) to 0.9 in steps of 0.1. Performance for each combination was estimated using 10-fold cross validation and averaging the performances on the validation set across the 10 iterations [57]. Once the hyper-parameters *N_HIDDEN_* and *η* were fixed, the MLP network was trained using the complete training set.

After the MLP was trained, results were evaluated. If the MLP output exceeded a threshold *Th*, the image was considered as having adequate quality (positive). Classification performance was evaluated in terms of sensitivity (*Se*), specificity (*Sp*), accuracy (*Acc*), positive predictive value (*PPV*), and *F*-Score (*F*_1_) [62]. It should be noted that these metrics depend on the value of *Th*. We obtained the receiver operating characteristic (ROC) curve to find the optimum value of *Th* in the training stage [62]. The optimum point of operation was selected during training as the point in the ROC curve closest to (0, 1), and the corresponding *Th* was used for classification in the test set. The area under the curve (*AUC*) was calculated to measure the robustness of the classifier [62]. The closer the *AUC* value is to 1, the better the classification performance and robustness are [62].

### 4.2. Feature Selection Results

**Figure 5:**
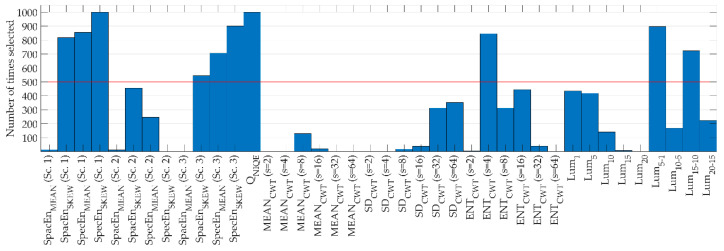
Results of fast correlation-based filter feature selection with a bootstrap approach. Features selected a number of times greater than 500 (red line) were included in the reduced subset.

The FCBF algorithm was applied over 1000 bootstrap replicates of the features extracted from the training set. Features selected on at least 500 runs of the bootstrap method were finally included in the reduced subset of optimum features for the classification task. Results of the feature selection stage are summarized in Figure 5. A total of 10 features formed the reduced subset. It should be noted that features from all proposed feature extraction methods were selected—six features from SSEQ (*SpacEn_SKEW_*, *SpecEn_MEAN_*, and *SpecEn_SKEW_* in the scales 1 and 3), the NIQE index *Q_NIQE_*, one from the CWT (*ENT_CWT_* at *s* = 4), and two from the luminosity analysis (*Lum*_5–1_ and *Lum*_15–10_).

### 4.3. Classification Results

**Figure 6:**
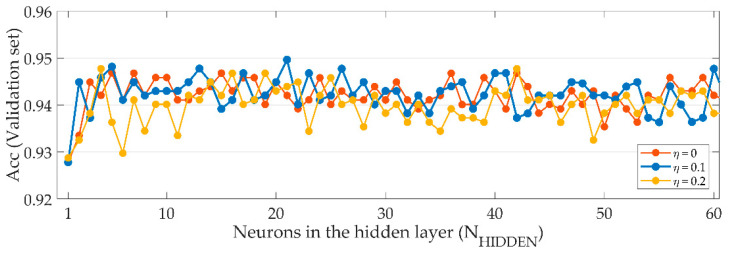
Estimated accuracy (*Acc*) for each combination of the number of neurons in the hidden layer (*N_HIDDEN_*) and the regularization parameter (*η*) using 10-fold cross validation. Regularization parameters *η* = 0 to *η* = 0.2.

The MLP configuration (*N_HIDDEN_* and *η*) had to be optimized in the training stage. Figure 6 represents the estimated performances for different combinations of *N_HIDDEN_* and *η*. Maximum *Acc* was reached with *N_HIDDEN_* = 21 neurons and *η* = 0.1. Therefore, this combination of hyper-parameters was used to obtain the final results on the study.

Once the hyper-parameters were fixed, the MLP network was trained using the whole training set. The optimum output threshold for the MLP was subsequently obtained using the ROC curve approach. We found that, with our training data, the optimum threshold was *Th* = 0.5 (*AUC* = 0.9803).

Once all the parameters of the classification stage were fixed, the final results of the proposed method were obtained for a new set of unseen images (test set). Table 1 summarizes the results for the test set in terms of *Se*, *Sp*, *Acc*, *PPV*, and *F*_1_.

## 5. Discussion

In this study, a novel RIQA method was proposed. It was based on generic features extracted from two different general-purpose NR-IQA methods, the CWT, and the luminosity of images using the HSV color model. Features were selected and subsequently classified using the FCBF algorithm and a MLP neural network, respectively. Results were obtained on a database of 2107 fundus images, reaching *Se* = 92.04%, *Sp* = 87.92%, *Acc* = 91.46%, *PPV* = 97.88%, and *F*_1_ = 0.9487 on the test set.

### 5.1. Preprocessing

A preprocessing step was included to enhance the images in our database and to improve the results in the subsequent processing stages. This preprocessing method has been successfully applied in previous studies for retinal vessels segmentation [40,63]. However, in previous studies, the FOV extension did not cover the whole area of the image [40,63]. In this work, we propose a novel modification of this FOV extension algorithm in order to cover the whole image. This approach has advantages for subsequent retinal image processing algorithms. One of them is that we could employ SSEQ and NIQE methods to analyze all the areas in the image before the block selection step [34,35]. Furthermore, the preprocessing method prevented border effects when the CWT and the Gaussian filter employed in luminosity analysis were applied. The absence of border effects increased the robustness of the features analyzed in this study.

### 5.2. Feature Extraction

After preprocessing, four different feature extraction methods were applied to characterize the images. A total of 40 features were extracted using SSEQ, NIQE, CWT, and luminosity in the HSV color model. Feature selection was subsequently applied to discard redundant or irrelevant features using the FCBF algorithm [56].

The complete set of features included 12 features from SSEQ method. To the best of our knowledge, SSEQ method has not been previously applied in RIQA. Measurements of *SpacEn* and *SpecEn* through the blocks of the images provided relevant information, especially in scales 1 and 3. Specifically, measures of *SpacEn_SKEW_*, *SpecEn_MEAN_*, and *SpecEn_SKEW_* for these scales were selected by FCBF to form the final feature subset. However, we observed that *SpacEn_MEAN_* was not selected. This may indicate that *SpacEn_MEAN_* was found redundant with respect to other features. Regarding the SSEQ method, we found that *SpacEn_SKEW_* and both *SpecEn*-derived features can be useful to distinguish images of adequate quality from inadequate quality ones. This result is consistent with other studies that successfully employed entropy-based measurements in the context of RIQA [20,24,26], biomedical signal processing [41,42], and strategic decision making [43].

A naturalness feature derived from the NIQE method was also considered. The quality feature *Q_NIQE_* is relevant for quality assessment since it was selected in all the bootstrap runs of the FCBF algorithm, together with *SpecEn_SKEW_*. Therefore, *Q_NIQE_* is complementary to all other features proposed in this study. Our results correlate with previous studies [7], where the NIQE index was used in combination with illumination and structural-based methods for RIQA.

Another set of features was extracted using the CWT. This method has been previously used to perform retinal vessel segmentation tasks and to discriminate healthy and diseased retinal images [40,46,63]. We calculated the CWT representations for retinal images at six scales using the Mexican hat mother wavelet. This wavelet was useful to identify sharp edges at varying scales, which are indicative of sharp images. As seen in Figure 3, representations for *s* = 4–16 showed stronger responses along thin-to-thick blood vessels. Sharp edges were found mainly in the OD and blood vessels and were associated with stronger CWT responses. Previous works that used the wavelet transform also stated the usefulness of this technique to assess the sharpness on fundus images [6,26,27]. In our study, sharpness of CWT representations was assessed with three novel features based on Shannon’s entropy and local variance, showing promising results. Characterization of the CWT representations was comprehensive, but these features were found redundant and only one of them (*ENT_CWT_* at *s* = 4) was finally selected using the FCBF algorithm.

Finally, luminosity features were also included in the analysis. This type of feature has been previously used to identify poorly illuminated fundus images. Luminosity of retinal images was widely studied using different color models, such as RGB, YUV, or L*a*b* [7,20,22]. In this study, we have found that the HSV color model is also useful to characterize poorly illuminated fundus images. The HSV color model allowed us to represent the luminosity of retinal images independently of their color. The color of retinal images is closely associated to the physical features of the patients (such as skin or iris color) and also to the acquisition process [26,52]. Therefore, separating the luminosity and chromatic information of the image is useful to study brightness-related features. We observed that the luminosity channel of the HSV color model performed better than illumination components of YUV and L*a*b* color models for the images in our database. More specifically, we found that the differences between light and dark areas were more prominent in the *V* color channel of HSV. This finding can be due to the better separation of the color information in the hue and saturation channels from the luminosity (*V*) [52]. Therefore, the *V* channel was less dependent on the color of retinal images. Previous studies have also employed the HSV color model in order to assess retinal image quality [26]. In that study, color information was more relevant and was extracted from a modified saturation channel. Alternatively, we found relevant features derived from luminosity contained in retinal images. We analyzed several features related to luminosity percentiles and their differences extracted from the *V* channel. Two of these features (*Lum*_5−1_ and *Lum*_15−10_) were selected by the FCBF algorithm, which indicates the relevance of luminosity features for RIQA.

### 5.3. Feature Selection and Classification

After the feature extraction stage, we used the FCBF feature selection method to obtain a subset of relevant and non-redundant features. Besides, we combined FCBF with bootstrapping to improve the robustness of this stage. To the best of our knowledge, this feature selection approach has not been previously used in RIQA methods. A reduced set of 10 features was finally selected by FCBF. This reduced set included features of the different feature extraction approaches analyzed in this study, which demonstrates that the proposed features are complementary. The general-purpose NR-IQA methods used in this work were found useful to characterize the quality of the images. Moreover, the CWT approach was found appropriate to assess the sharpness of the retinal blood vessels and the OD. Finally, the HSV color model was useful to assess the luminosity of images.

In the classification stage, a MLP neural network was used. The three-layer MLP had 10 neurons in the input layer, *N_HIDDEN_* = 21 neurons in the hidden layer, and 1 neuron in the output layer to perform binary classification. To achieve good generalization and avoid overfitting during training, we set the regularization parameter to *η* = 0.1. These parameters reported the maximum estimated *Acc* using 10-fold cross validation, as shown in Figure 6. Higher values of *N_HIDDEN_* did not improve the results, while other values of *η* reported slightly lower performance. As only about 14% of the images in the database used in this work had inadequate quality, we had to deal with class imbalance. Synthetic samples of the minority class were obtained using SMOTE, and were used for MLP training. Using this technique, we reached almost balanced *Se* and *Sp* values while *PPV* and *F*_1_ were also high (Table 1). This tradeoff between *Se* and *Sp* indicates that our neural network implementation was able to model the particularities of both good quality and bad quality images.

### 5.4. Results

Results on the test set achieved *Acc* = 91.46% and *F*_1_ = 0.9487. The latter measure is especially relevant since *F*_1_ represents the tradeoff between a high detection rate (*Se* = 92.04%) and high probability of correct detection (*PPV* = 97.88%). Besides, the vast majority of images were correctly classified, while an acceptable tradeoff between *Se* and *Sp* was reached. Our results showed 18 false positives, reaching *Sp* = 87.92%. We also obtained 72 false negatives out of the 1054 images in the test set. Our RIQA method failed to classify images that did not perfectly fit the ideal characteristics of adequate or inadequate quality images. Misclassifications were frequent when images did not have a perfect focus or when a dark but partially sharp image was analyzed. It is important to note that, if this RIQA method was included as the first stage of a more general retinal image analysis algorithm, inadequate quality images misclassified using the proposed method would be further processed. This issue may influence the results of the subsequent image analysis algorithms. Conversely, when an adequate quality image is misclassified, the image would be rejected by the RIQA algorithm. In clinical settings, this can be inconvenient because the photographer would need to capture an adequate-quality fundus image again. However, this issue is not likely to have an important influence over the results of a more general retinal image analysis method on the image.

**Figure 7:**
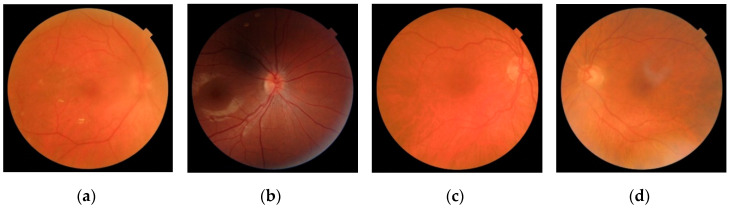
Examples of original images in our database that were misclassified using the proposed method; (**a**,**b**): False positives; (**c**,**d**): False negatives.

Some examples of misclassified images in our study are shown in Figure 7. In Figure 7a, the OD and the main arcades are blurred. However, the vessels and some bright retinal lesions (exudates) are reasonably sharp in other areas of the image. In the case of Figure 7b, the image has some dark areas, but the rest of the image is sharp. These two images were incorrectly classified as adequate quality images. The cases shown in Figure 7c,d are examples of false negatives. Both images appear slightly blurred due to poor focus or artifacts, respectively. Thus, the automatic method considered them as inadequate-quality images. However, human graders considered that they had enough quality to be analyzed. These examples reveal that quality assessment of fundus images is a challenging task, that may be influenced by the subjectivity or experience of human graders [22].

Our final results were similar to those in previous studies. However, comparisons should be made with caution since results are generally measured on different databases and with varying metrics. We evaluated the results of the proposed method using the test set of our database, formed by 1054 images. The majority of studies presented their results using *Se* and *Sp* and, in some cases, *Acc*. In this work, we also included *PPV* and *F*_1_ in order to better assess the performance of the proposed method. However, these measures are not commonly used in this context. Structural and generic methods achieved *Se* and *Sp* around 90%, and *Acc* was over 90% in most cases. Among structural methods, Fleming et al. [8] reached *Se* = 92.60% and *Sp* = 90.00% with 98 images. Other authors [11] validated their method using 400 images, reaching *Se* = 95.33% and *Sp* = 91.13%. Wang et al. [22] achieved *Se* = 87.45% and *Sp* = 91.66% using 536 images, while Abdel-Hamid et al. [27] reached *F*_1_ = 0.8780 using 190 images. Other authors also combined the NIQE index with structural and illumination features, reaching *Acc* = 93.60% with 194 images. The best performance among generic methods was achieved using the MLP neural network [12]. Results reached *Se* = 99.49% and *Sp* = 99.76% in a database formed by 848 images [12]. Hybrid approaches also showed remarkable results, although they are more complex than generic methods [5,29]. Paulus et al. [5] employed 301 images, achieving *Acc* = 91.70%. In other studies, *F*_1_ reached 0.9960 using 194 images [29]. Deep learning-based methods have been previously used for RIQA, achieving excellent performances. Among them, Reference [30] should be remarked, since it achieved a perfect classification over 3425 images. However, it should be noted that only 123 (3.6%) of these images corresponded to inadequate quality images. Therefore, class imbalance should be taken into account when analyzing these results. In this work, novel generic features were proposed. We achieved similar results compared to other generic methods, suggesting that our novel features form a viable alternative.

### 5.5. Limitations and Future Work

Our study has some limitations that should be pointed out. The database employed in this work comprised 2107 fundus images, but only 297 of them (14%) were labeled as inadequate quality images. This proportion is similar to other studies, but the number of examples available for training and testing may not be large enough. To overcome this issue, we used SMOTE to obtain a more balanced dataset for training. Additionally, despite the fact that the size of our database was higher than the ones used in other studies [12,20,29], it would be desirable to increase the number of available images and to investigate alternative techniques to deal with class imbalance in future studies. We also found that quality assessment by human graders is a challenging task and is not free from subjectivity. Although the rules for quality assessment were fixed beforehand, uncertainty may appear in cases where focus or illumination are uneven. In future studies, we would try to extend the quality assessment rules in order to cover the doubtful cases. Finally, in order to improve our results in the future, we would like to investigate alternative features and classification methods, including deep learning-based approaches.

## 6. Conclusions

The main objective of this study was to combine novel generic features for automatic RIQA. We found that SSEQ and NIQE methods can be useful to assess retinal image quality, and can be complementary with sharpness and luminosity features. Specifically, we found that *Q_NIQE_* and novel *SpacEn* and *SpecEn* derived features were relevant for this task. These features were successfully combined with *ENT_CWT_* and luminosity-related measurements, showing their complementarity. Our results suggest that this algorithm can be an important first step in more complex computer-aided diagnostic systems aimed at detecting ocular diseases like DR or AMD.

## Figures and Tables

**Table 1:** Results of the multilayer perceptron (MLP) neural network on the test set.

*Se* (%)	*Sp* (%)	*Acc* (%)	*PPV* (%)	*F*_1_
92.04	87.92	91.46	97.88	0.9487

*Se*: Sensitivity; *Sp*: Specificity; *Acc*: Accuracy; *PPV*: Positive Predictive Value; *F*_1_: *F*-Score.
